# Reconstructing radial stem size changes of trees with machine learning

**DOI:** 10.1098/rsif.2022.0349

**Published:** 2022-09-21

**Authors:** Mirko Luković, Roman Zweifel, Guillaume Thiry, Ce Zhang, Mark Schubert

**Affiliations:** ^1^ Empa, Swiss Federal Laboratories for Materials Science and Technology, Laboratory for Cellulose & Wood Materials, Group WoodTec, 8600 Dübendorf, Switzerland; ^2^ WSL, Swiss Federal Institute for Forest, Snow and Landscape Research, 8903 Birmensdorf, Switzerland; ^3^ ETH Zurich, Department of Computer Science, 8092 Zürich, Switzerland

**Keywords:** time-series analysis, imputation, tree growth, machine learning, convolutional neural networks, long short-term memory

## Abstract

Like many scientists, ecologists depend heavily on continuous uninterrupted data in order to understand better the object of their study. Although this might be straightforward to achieve under controlled laboratory conditions, the situation is easily complicated under field conditions where sensors and data transmission are affected by harsh weather, living organisms, changes in atmospheric conditions etc. This often results in parts of the data being corrupted or missing altogether. We propose the use of the most recent machine-learning techniques to reverse such data losses in multi-channel time series. In particular, we focus on tree stem growth data obtained from the TreeNet project, which monitors the changes in stem radius and environmental conditions of a few hundred trees across Switzerland. In the first part of the study, we test the performance of five architectures based on encoders and recurrent and convolutional neural networks, and we show that a deep neural network combining long short-term memory with one-dimensional convolutional layers performs the best. In the second part, we adopt this model to reconstruct the original TreeNet dataset, which we then use in a separate classification problem to show the effect of the proposed gap-filling procedure.

## 1. Introduction

Turning automatically measured stem radius (SR) data into biological information related to tree responses to environmental conditions is a well-established procedure [1–4]. It involves cleaning the raw datasets, processing and separating it into growth and tree water-related variations before being analysed in terms of species-specific responses [5]. This is one of the principle tasks of the *TreeNet* project, a Swiss research network in which automated tree SR fluctuations measured with *point dendrometers* are analysed in terms of forest ecosystem responses to climate change [6]. It runs a fully automated data acquisition system with a central database and is focused on the development of new methods that will accelerate data processing to help us obtain adequate tree physiological answers faster, e.g. in the case of a drought event like in the summer of 2018. Machine-learning algorithms have a great potential to do so [7].

Nevertheless, a common and very frequent problem with time-series data is the presence of missing and corrupt values, especially if it is obtained through an automated process such as the TreeNet project. While this might not be a problem when there are occasional time stamps or smaller gaps with missing or corrupt data, the issue becomes serious in cases where the gaps are frequent and of the order of days. According to recent studies, intermittent growth with frequent changes between days with and without growth has been observed in tree stems [4]. In this particular example, much valuable information could be lost with only a few data gaps due to the intermittence of growth and the associated shorter continuous growth periods. The common methods used for dealing with this problem, known as gap-filling or imputation, range from simple techniques such as interpolation or removal of the missing parts to more advanced methods such as regression and machine learning. Methods that use smoothing, interpolation and splines are simple and efficient, but they do not capture variable correlations and often miss complex patterns when dealing with imputation [8]. For this reason, we explore the effectiveness of using recurrent and convolutional neural networks to reconstruct the missing values of multi-channel time series.

The aim of the study is to provide a robust and easy-to-use method capable of accurately reconstructing multi-channel time series so that more data becomes available for analysis.

In §2, we describe the characteristics of the TreeNet data. They include time-dependent measurements of the stem radii of trees and their atmospheric and soil conditions (table 1). A total of seven different variables from 190 trees were stored in separate channels of the time series. The time series ranged in length from 1 to 9 years. The trees belonged to 12 different species, most of which were well represented in the data with a few dozen trees each, but some with only two or three trees per species. To obtain a more balanced dataset, the four species with three or fewer trees were excluded, leaving 168 trees in the dataset.

**Table 1.** The properties measured over time for each tree. The SR was monitored using point dendrometers mounted on each tree. The meteorological and soil parameters were measured at each site and then attributed to the trees at the corresponding sites.

feature	abbreviation
stem radius (μm)	SR
temperature (${\;}^{\circ }C$)	temp
relative humidity (%)	rh
vapour pressure deficit (kPa)	vpd
solar radiation (W m^−2^)	rad
soil water potential (kPa)	swp
total precipitations (mm)	total precip

In §3, we focus on time-series reconstruction. Our goal was to recover the sections with missing data in each of the seven channels, paying particular attention to the SR data. Tree stem growth and water deficit are derivatives of these radius changes, and both are central to understanding how trees respond to environmental change [1–3]. We first present two simple and well-established regression methods, namely ridge and multi-layer perceptron regression, which were used as reference in the study. We then present the best five out of the different machine-learning architectures that we tested. They are all based on supervised learning and consist of autoencoders, long short memory (LSTM) and convolutional neural networks (CNN). These deep neural network models take into account auto-correlations over time and are therefore well suited for time-series analysis [8]. Overall, we compare the performance of seven different models.

In §4 we present the part of our study where we test the effect of data reconstruction on a classification problem. Here, we quantify the accuracy of classifying the time series into tree species before and after data reconstruction.

## 2. Data properties and preparation

**Figure 1.**
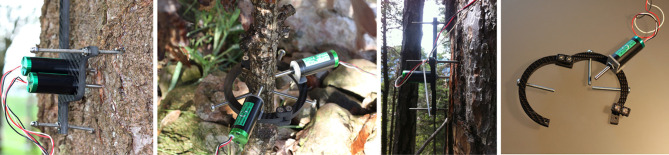
Collection of point dendrometers [9]. The carbon frames are either T-shaped (for large stems) or O-shaped (for small stems or branches) and are anchored in the stem with stainless steel rods. Up to three sensors are attached to the different type of frames in order to measure different expositions at the stem or to measure stem radius fluctuations over bark and on the xylem separately [6].

The main signal that we dealt with in the current study was the change in the SR of each tree. The stem change was continuously monitored at an effective resolution of 1 μm at 10 min time intervals using automated point dendrometers placed at the stem of each tree at breast height (figure 1) [10]. Although the time resolution of the sensors was 10 min, the data that we used for the study was coarse grained by averaging to an hourly resolution. Alongside the SR data, we also included signals registered by weather and soil sensors close to the tree sites, shown in table 1. Each of these signals appears as a channel in the overall time-series studied.

**Figure 2.**
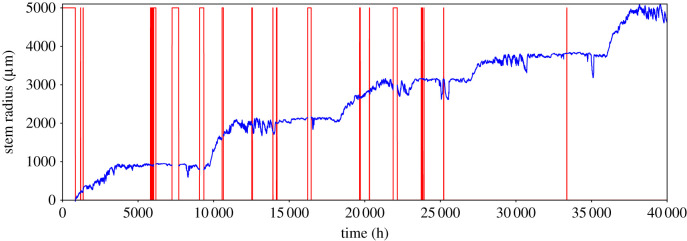
SR time series (change in stem radius) of a single tree (blue curve), showing the presence of missing data (red lines).

**Figure 3.**
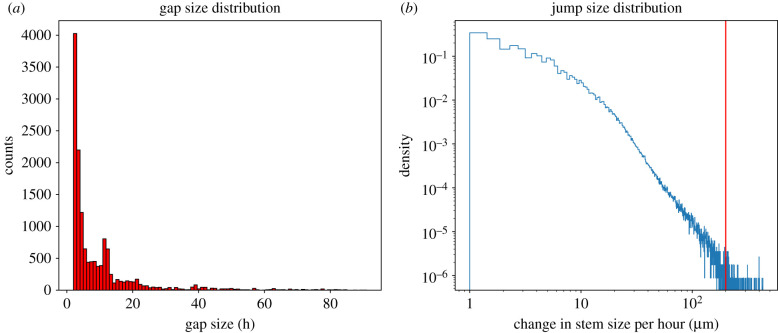
(*a*) Distribution of gap sizes (missing data intervals) extracted only from the stem radius (SR) channels of all the available time series. For a better visualization, the largest 5% of the gaps are not included in the distribution. From the distribution, we calculated that 95% of the gaps are below 80 h, and 90% are below 30 h. (*b*) Distribution of jump sizes in the stem radius channel of all the available time series. The vertical red line shows the cut-off point at 200 μm.

The fundamental problem with the data considered was that there were intervals of missing or corrupted values (gaps) present in the time series (see figure 2 for an example), which were registered as not a number (NaNs) in the data array. Most gaps were caused by minor problems in the acquisition and therefore only a few hours of data were missed. However, occasionally bigger problems caused acquisition to stop altogether, until the device was replaced or adjusted, perhaps days or months later (figure 3). As far as the point dendrometers and their loggers are concerned, defects due to mechanical effects, e.g. damage caused by rodents, water penetrating the electronics, or failed data transfer connections were among the most frequent causes. This caused data acquisition to either be erroneous or cease altogether. It is also important to mention that out of all the channels that were available, the one with most missing data was the SR channel, accounting for around 75% (table 2) of the total number of time stamps with missing data. Although there were rather long gaps of data in the time series, most of them were short, as illustrated by the distribution in figure 3. In addition, there was little to no correlation between the time of occurrence of missing SR data and missing weather or soil data. In other words, whenever the SR channel was interrupted, it was certain that data for at least one of the other channel was being registered (this can be seen from table 2). The reason for this is because the data came from independent measuring devices. This meant that we could also rely on cross-correlations between different channels in order to reconstruct the SR signal.

**Table 2.** The table shows how the missing data is distributed across the channels. There are time stamps where data of only one channel is missing, but there are also many where data is missing from two or more channels. For example, 53.5% of all missing data falls on the SR and swp channels.

channels with missing values	no. time stamps with missing data	share within missing data (%)	proportion of total time stamps (%)
SR, swp	303 595	53.46	4.65
swp	134 210	23.63	2.06
SR	124 438	21.91	1.91
total precip	3374	0.59	0.05
SR, rad, swp	1770	0.31	0.03
rad	198	0.03	0.00
temp, rh, vpd	176	0.03	0.00

Total number of time stamps in the dataset is 6 528 585

Since our main objective was to train a deep neural network to replace missing values and therefore expand the amount of available data, we first had to make sure that it was cleaned and converted to the correct format as input for the various algorithms used.

We then proceeded to eliminate any unrealistic changes in the SR values over the minimum time difference of 1 h. A distribution of these changes or jumps in SR between consecutive hours, across entire dataset consisting of 168 time series, is shown in figure 3. In order to eliminate outliers, we ignored values greater than 200 μm, which were assumed to be unrealistically high and probably induced by external mechanical disturbances (less than 2% of data).

## 3. Tree stem data reconstruction

Most machine-learning methods have generally a well-defined output format such as a class, a number or an action to be determined. However, in our case, we are dealing with variability of the gap sizes, their position in the time-series segments and the features affected. In principle, one cannot use simple regression on the known values of a particular channel in order to predict the missing interval. Rather, it is necessary to predict the entire sequence of values of that particular channel based on the data available in other channels. Therefore, there is a need for a more general approach, one that would cover more cases of missing data and that can tackle a finer time resolution, while dealing with all channels at the same time.

With neural networks, the above-mentioned limitations can be overcome. It is in fact possible to train a deep neural network such that it can make use of all the channels, including the ones that are corrupted. The entire multi-channel time series can be loaded as input so that a copy comes out with any existing gaps amended. The process of correcting the time series in this case is akin to the *inpainting* technique used in restoring damaged paintings and more recently also digital media [11]. Inpainting is the process where damaged, deteriorating or missing parts of an image or artwork are filled in to present a complete version [12,13].

To get a reference for the more complex neural network models, we first used two well-established algorithms, namely ridge regression and multi-layer perceptron regression. We will refer to them as the *baseline* models. Subsequently, we constructed different models based on LSTM and convolutional neural networks, which we will refer to as *test* models. Out of all the test models that we created, we present the results of the best five in terms of reconstruction accuracy.

We extracted 30-day uninterrupted and non-overlapping segments from the original TreeNet dataset, avoiding the NaNs so that there were no missing values in any of the seven channels. We tried different segment lengths, between 10 and 60, and found that 30-day segments were the best trade-off between the accuracy of the results and the length (longest) of the segment. The segments were then normalized between 0 and 1 and then duplicated. One copy of this data was kept the way it was, without any missing data, and was used as the ground truth for the model training and testing phases. These constituted the labels in the context of supervised learning. In the second copy, data gaps were created in randomly selected channels and segment sections and used for the learning and testing phases of model development. The gaps were all the same length (10 days) and could occur in more than one channel of the same segment. To maximize learning, we ensured that in each segment the SR channel and at least one other channel had a data gap. Twenty per cent of the data in both data copies was set aside and used for testing at the very end (test set). Eighty per cent of the data was used for training and validation. The split was made in such a way as to preserve the correspondence between the labels and the input. The resulting split was then used for both the base models and the test models. While the same test set was used to evaluate the performance of each model at the end, the split between training and validation was randomized from model to model. To avoid bias in the analysis results, the data had to be distributed among the training, validation and test sets in such a way that all segments belonging to the same tree were in the same dataset.

### 3.1. Methods: baseline models

**Figure 4.**
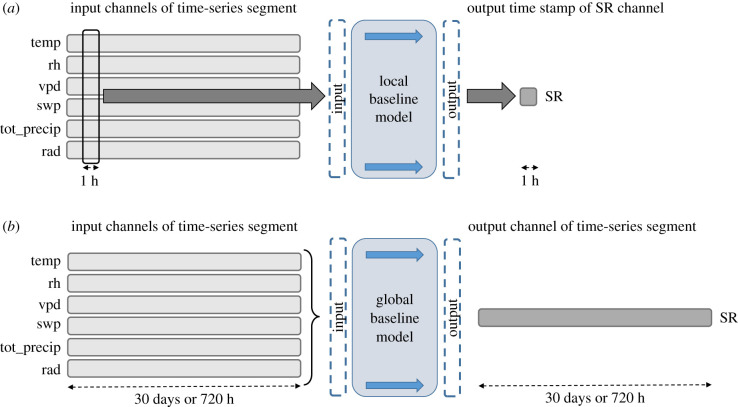
(*a*) Reconstruction using the local baseline models. This method takes a single time stamp across multiple channels as input and produces a single time stamp of the stem radius. This is done multiple times until the desired stem radius sequence is reconstructed. The input must be free of missing data. (*b*) Reconstruction using the global baseline models. This method takes multiple channels as input and predicts the entire stem radius channel of the segment (shown in a darker shade of grey compared with the input). The input must be free of missing data.

For the two baseline regression models, we only used the clean data, without the artificially created gaps (first copy). The objective was to create a regression model able to predict the complete sequence of the SR values using the rest of the channels as input. We performed the fitting using two different approaches for each of the regression methods, meaning that we had a total of four baseline models in the end. The first approach, which we call *local*, consisted in preparing the data so that every time stamp was a sample, where the input was an array of seven values, one for every feature (except the SR) and where the label was the value of the SR for that particular time stamp. The locality of the approach stems from the fact that we were using information from only one time instant to predict the SR at the same point in time. This approach is illustrated in figure 4. We will refer to the second approach as *global*, where each sample was represented by an entire segment of 30 days. The input was much larger in that case and it consisted of all the values of the seven channels, each having 720 values, excluding the SR channel. In this situation, temporal correlations of the sequence, understood as a global property, were taken into consideration (figure 4). In what follows, we will use the baseline models in combination with global and local properties.

The baseline models were implemented using the Scikit-Learn Python library [14]. They both made use of regularization parameters, which we had to choose. We tried systematically a range of different values, but the result did not change much with respect to the default parameter. The multi-layer perceptron regressor has a few other additional hyperparameters common with neural networks, the most important being the number of layers, number of neurons per layer, learning rate, regularization factor and early stopping time. We started with the default parameters provided by the library and then used fine-tuning to obtain our final result.

### 3.2. Methods: test models

The following step was to choose an adequate deep neural network architecture that could learn how to fill the gaps of missing data. For this task, we explored models that were based on three fundamentally different architectures used in the machine-learning community, namely long short-term memory (LSTM) networks, one-dimensional convolutional neural networks (CNN) and autoencoders.

LSTM cells are proven to be very efficient in dealing with time-series data, and the encoder–decoder architecture is often used for reconstruction tasks. LSTM networks fall under the broader range of recurrent neural networks (RNNs), which are used for processing sequential data [15]. These networks make use of feedback connections in order to process entire sequences of data, which can also be of variable length [16]. In addition, RNNs have several important properties such as strong prediction performance as well as the ability to capture long-term temporal dependencies and variable-length observations [8]. LSTM networks are more complex and sophisticated compared with ordinary recurrent neural networks. The LSTM units or neurons also contain cyclic connections, but in addition they have a memory cell and logistic gates (write, read and forget) that help better regulate the flow of information. The network can be made more versatile by putting two LSTM layers together. One serves as an encoder and the other as a decoder. The advantage in this case is that the dimensions of the input and output do not have to be the same, a useful trait when using neural networks in language translation for example [17].

One-dimensional convolutional neural networks are similar to recurrent neural networks in that they can share parameters across time. The convolution operation relates a small neighbourhood of the input to a point in the output sequence [16]. Additionally, convolutional layers provide translational invariance for the patterns detected within the sequences.

An autoencoder consists of multiple neural layers (very often CNNs) that reduce the dimension of the presented data down to a middle layer, also known as the latent layer. Subsequently, the information in the latent layer is fed forward and processed by another set of neural layers, expanding it back to the original sequence dimensions [18]. The autoencoder learns how to store a representation of the input data in a space of smaller dimensions. The dimensionality reduction ‘forces’ the autoencoder to ignore any insignificant data such as noise. What makes this method suitable for our study is the fact that we can consider the missing values in our data as the noise in the input.

**Figure 5.**
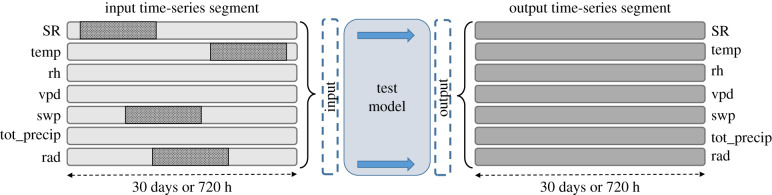
Reconstruction of the time series using deep neural networks. These methods can deal with data gaps across multiple channels. As input, we have a multi-channel time series with 10-day data gaps in it (darker sections). As output, we obtain a reconstructed time series in which all values are predicted, not just those that were missing. In the image, this is represented by a darker shade of grey in the output. Since the entire sequence of data for each channel is used as a single input, the model is certainly global in the sense described in the text.

A diagram of the relationship between the input and output derived from the multi-channel time series can be seen in figure 5. The five test models were all trained to process the input data and predict the values for all the time stamps and not just those that correspond to the gaps. Therefore, all the values that we obtained in the output were estimates, including those that were not missing initially. Following the inpainting technique, we substituted all the gaps in the input with the corresponding predicted values to obtain the reconstructed signal.

Each of the proposed models described above had to be assigned hyperparameters and trained before they could be used in any form of gap-filling. In table 3, we list the hyperparameters that we used for each of the models. We started the hyperparameter selection by choosing a single hidden layer with a random number of neurons of the order of the input size. We chose the rest of the hyperparameter values by trial and error. From there, we used a range of values to systematically tune the hyperparameters. For each of the five models, we used the mean squared error (MSE) as the loss function, which is necessary in the training phase to give a quantitative evaluation of the comparison between the predicted values and the ground truth. Therefore, once the loss stopped diminishing across the training epochs, we concluded the search for hyperparameters. Furthermore, we used an *adam* optimizer throughout our study with dropout regularization, and *early stopping* in the training phase in order to avoid overfitting problems. Finally, in order to distinguish better the main signal from the missing data, the time stamps corresponding to the missing values were assigned values of −1 (note that the signal is normalized so that all the values fit in the interval [0,1]). All the models were created and trained using Python and the TensorFlow machine-learning platform [19].

**Table 3.** Hyperparameters tuned to optimize each of the deep neural models.

model	hyperparameters tuned
LSTM	#layers, #neurons, dropout rate, regularizers:L1,L2
LSTM + CNN	#layers, #neurons, #filters, dropout rate
LSTM + encoder	#layers, #neurons, dropout rate
Autoencoder 2D	#layers, #neurons, #filters dropout rate, stride length
LSTM + autoencoder	#layers, #neurons, #filters, dropout rate

### 3.3. Results and discussion of data reconstruction

**Figure 6.**
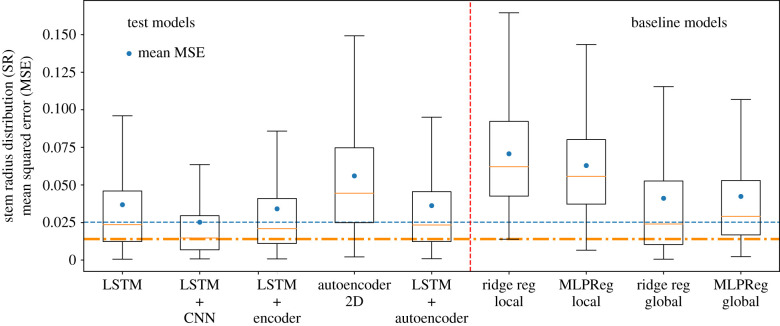
Comparison of the accuracy of the different algorithms used to fill the artificially created gaps in the data. The box plots represent the distribution of the mean squared errors (MSE) between the predicted gap values of the stem radius (SR) and the ground truth. Only the data gap of each time-series segment was used for determining the MSE. Each box extends from the first quartile (Q1) to the third quartile (Q3) of the data, with a line at the median. The whiskers extend from the box by 1.5 times the inter quartile range (IQR). The outliers are not shown. On the left side of the dashed vertical line are the box plots that correspond to the deep neural models. On the right side are the ones related to the baseline models. The horizontal dot-dashed orange line indicates the median MSE related to the LSTM + CNN model and the horizontal dashed blue line corresponds to the mean MSE related to the LSTM + CNN model.

We present here the performance results of the baseline models and our test models. Using the test set that was not used for the training phase, we were able to determine the accuracies of these models. They are represented in terms of the MSE between the ground truth and the predicted values. It is important to emphasize that the MSE was calculated using only the gaps and not the entire segments. The results corresponding to the baseline models are shown in the right part of figure 6. Out of the four baseline models, the global variant of ridge regression performed the best.

Regarding the test models, we tried different combinations of long short-term memory networks, autoencoders and convolutional neural networks. The architecture and other details of each of the models are presented as graphs with a description in the appendix (figures 11–15). In figure 6, we demonstrate the performance of the best five of the various combinations that we tried. The box plots in figure 6 show how the MSE of the samples (30 h segments in this case) are distributed for each of the models. The most accurate model turns out to be the one that combines two LSTM layers in the encoder–decoder form, coupled with two one-dimensional convolutional neural networks, i.e. LSTM + CNN. On a more general note, the results also demonstrate that models having a combination of architectures (LSTM + CNN, LSTM+encoder and LSTM+autoencoder) all perform better than models with a single one (LSTM and Autoencoder2D). We also tried a few neural networks with only convolutional layers and a few with only dense layers, but the RMS (accuracy) values were much higher than all the others, so we decided not to include them at all. From our results, we conclude that it is better to combine the architectures discussed rather than using them on their own.

**Figure 7.**
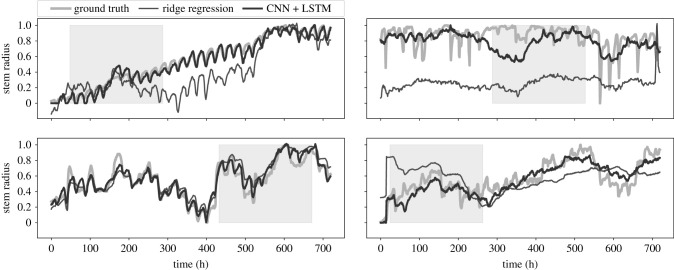
Examples of reconstructed stem radius channels of randomly chosen samples. The 10-day artificially created gaps are inside the grey shaded area. Results are shown for ridge regression (global) and the CNN + LSTM combination. Shown are the reconstructions of the entire time-series segment, and not just the gaps.

In figure 7, we present a few representative examples of the SR channels, qualitatively comparing the predictive capabilities of the CNN + LSTM model compared with global ridge regression. It shows that both models, especially CNN + LSTM, are able to predict the trends of the signal. This is a strong indicator that the cross-correlation among the seven channels plays an important role for predicting the missing values. The CNN + LSTM is significantly better at reproducing the cycles that are observed in the SR dynamics.

**Figure 8.**
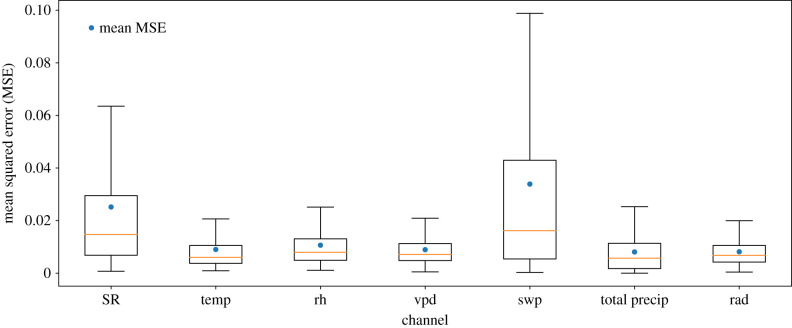
Reconstruction accuracy of the LSTM + CNN model for each channel separately. Presented are box plots of the MSE between the reconstructed gap values of each channel and the ground truth. Each box plot represents the MSE for a different channel.

**Figure 9.**
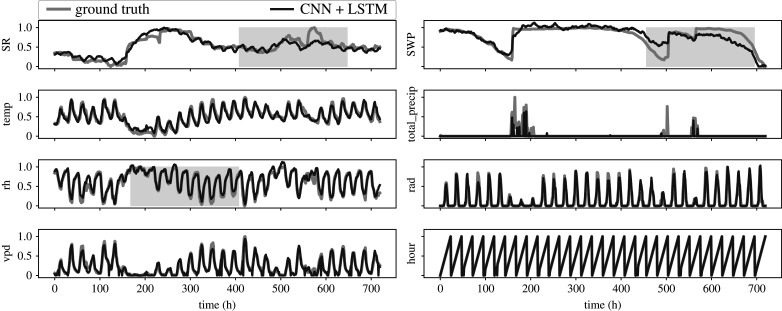
An example of a reconstructed time series using the LSTM + CNN model. All seven features and the time of day are shown. The shaded grey area is the interval where data was artificially removed.

As we mentioned above, the deep neural network architecture is not limited to the prediction of only one channel. On the contrary, it can be used to fill data gaps in two or more channels simultaneously. The accessible data within a corrupted channel is also used as information relevant for the prediction. In figure 8, we show the performance of the model with respect to each channel of the time series, and in figure 9 we exemplify the performance using a single sample of time series. They show that the SR and swp channels are the most difficult to reconstruct precisely. In both cases, the average and variability are much higher than for the other channels.

## 4. Classification with reconstructed data

In the second part of the study, we tested the best data reconstruction method from §3 using a simple classification problem. The task was to train a deep neural network to classify 30-day segments of TreeNet time series (input) according to the tree species (output) and determine whether time-series gap-filling makes a difference in accuracy. The rationale was that accurately reconstructed time series would provide more data for training the classifier, thus making it more accurate.

For training the classifier, we relied on the assumption that the growth patterns of stem radii, and therefore the corresponding time series, would be different from one species to another, especially when placed in the context of the climatic conditions surrounding each tree. For example, a difference in the growth periods of tree species or the presence of different growth rates due to different climatic conditions, would be reflected as different patterns in the stem radii time series. We decided to use the deep residual neural network (ResNet) for this classification task [20]. The model has been successfully used in many classification tasks, and its implementation is readily available for most deep learning platforms such as TensorFlow [21].

### 4.1. Methods: classification

The ResNet algorithm is based on a series of convolutional layers and residual connections between some of them, while the last part of the network consists of fully connected layers. In addition, each neuron is activated according to the rectified linear unit function. It is a common and widely used activation function whose purpose is to introduce nonlinearity into the output of the neuron. Our TensorFlow implementation of the classifier was a slightly modified version of the ResNet network used by Fawaz *et al.* [22,23]. In their study, the authors showed that the ResNet architecture had the best classification performance for the majority of time-series data that they tested.

Before training the ResNet model, we labelled each time series of the original TreeNet data (168 time series) according to the tree species that it belonged to. Thereafter, we divided the data so that 10% was reserved for the final testing phase, 80% for training and 10% for validation. The test set was prepared by extracting 30-day uninterrupted intervals from the time series, as already discussed in §3.

The train and validation sets were then used together with ResNet to construct two distinct deep neural models, the *standard* model and the *reconstructed* model. For the former case, we extracted 30-day uninterrupted segments from the train/validation time series, while in the latter case, the time series were reconstructed with the LSTM + CNN algorithm.

In the case of the reconstructed model, each train and validation time series was first divided into equal 30-day segments. If there were missing values, the segment was reconstructed, but only if less than two-thirds of the time stamps were missing. Otherwise, the entire segment was discarded. The choice of two-thirds was used to maximize the number of segments that could be repaired while always having enough data left for reconstruction. From figure 3, we can deduce that over 95% of the gaps are less than 4 days. Therefore, the LSTM + CNN model together with the two-thirds threshold resulted in a robust combination. The distribution according to tree species of the segments of the standard and reconstructed set are shown in table 4.

**Table 4.** Available species-specific tree data for the classification. The data segment length was 30 days.

tree species	no. tree samples	no. segments w/o reconstruction	no. segments w reconstruction	species label	species group
*Pinus sylvestris*	24	1086	1325	0	gymnosperm
*Fagus sylvatica*	42	1389	1939	1	angiosperm
*Picea abies*	53	2233	2783	2	gymnosperm
*Abies alba*	10	415	525	3	gymnosperm
*Quercus petraea*	10	396	535	4	angiosperm
*Quercus pubescens*	12	609	655	5	angiosperm
*Fraxinus excelsior*	7	284	351	6	angiosperm
*Quercus cerris*	10	250	303	7	angiosperm
total	168	6662	8416	—	—

The training was done over 300 epochs and was also used to determine the stopping time before overfitting became evident. Finally, we added some regularization to the network with batch normalization and dropout. Both models were initialized and trained from scratch 10 times using different train-validation splits of their respective data and then tested with the same test set. The aim was to obtain a more robust accuracy score by averaging the results.

Several basic models such as logistic regression or random forest were initially tested to establish a baseline on the classification (results not shown). However, the use of convolutional neural networks quickly proved to be the better method for this classification, therefore, we only present those results here. This is not really surprising as convolutions are able to take full advantage of the structure of the input: a time series is not so different from a one-dimensional image and convolutional layers in general perform really well on image classification [24,25].

### 4.2. Results and discussion of the classification

The goal of the classification experiment was to determine how well the proposed imputation method works in a real situation with real data. It is clear that being able to accurately classify segments of tree SR sequences according to the tree species might not be very useful to us, since we know beforehand which tree species each sequence belongs to. Nevertheless, we wanted to use this case study as an alternative and final assessment of the impact of the data reconstruction on the accuracy of classification.

**Figure 10.**
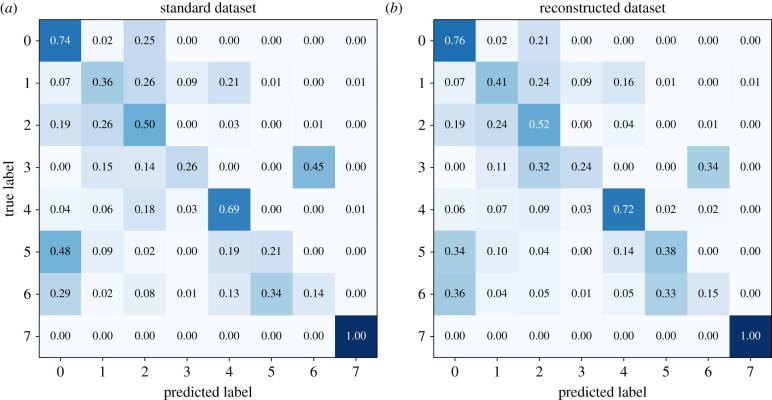
Confusion matrices of the ResNet classification model averaged over 10 realizations. Each realization consisted of a different split in the train-validation set. The matrix cells on the diagonal show the percentage of segments that were correctly classified according to its species. The interpretation of the labels is given in table 4. The off-diagonals show the percentage of false positives and false negatives. (*a*) Performance of the standard model with an average (taken over all the species) accuracy of 48%. (*b*) Performance of the reconstructed model with an average (taken over all the species) accuracy of 52%.

**Figure 11.**
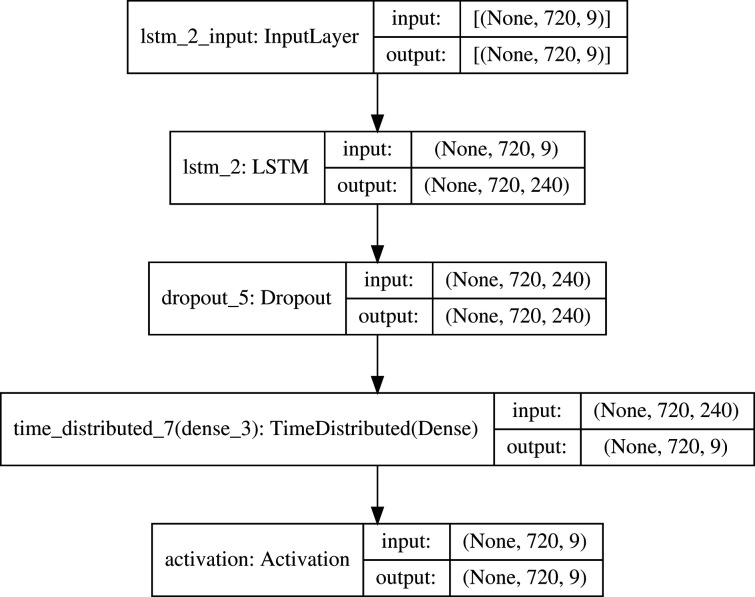
LSTM: a network with a single LSTM layer with an additional dense layer before the output.

**Figure 12.**
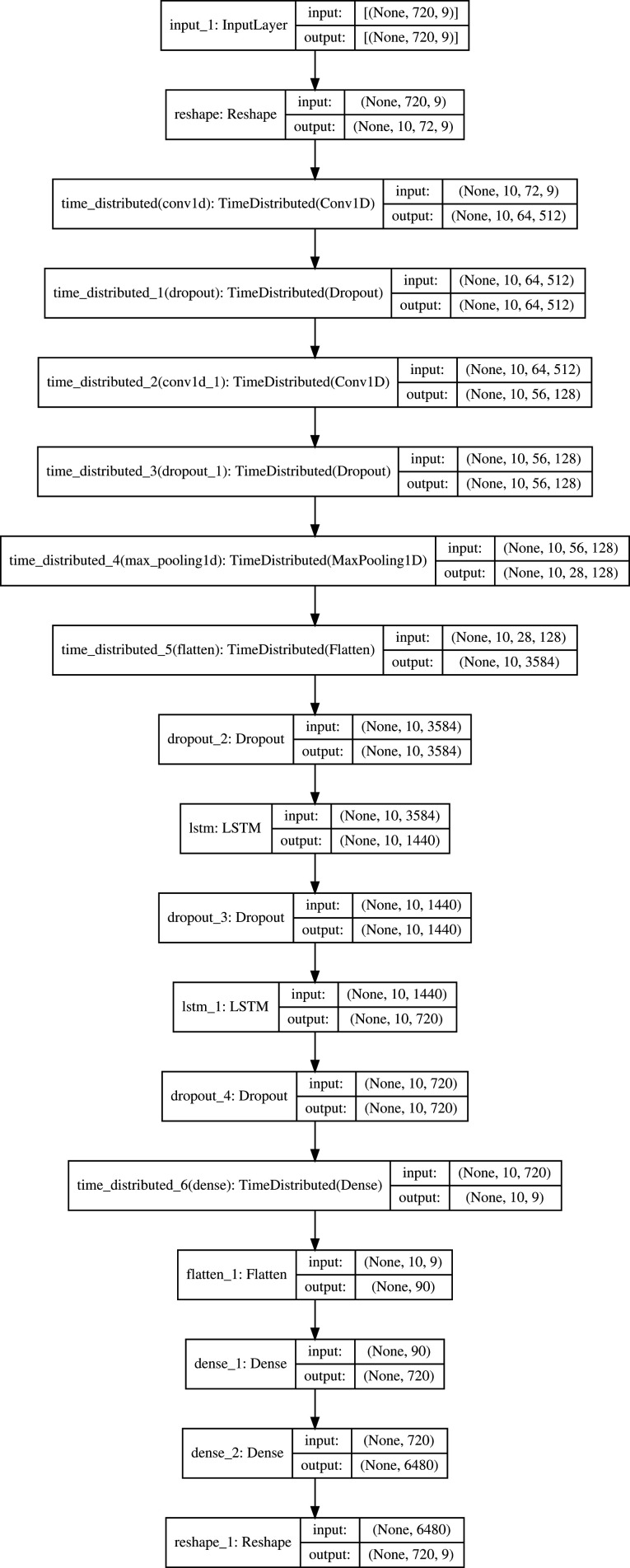
LSTM + CNN: two CNN layers combined with an LSTM encoder layer and an LSTM decoder layer with additional dense layers before the output.

**Figure 13.**
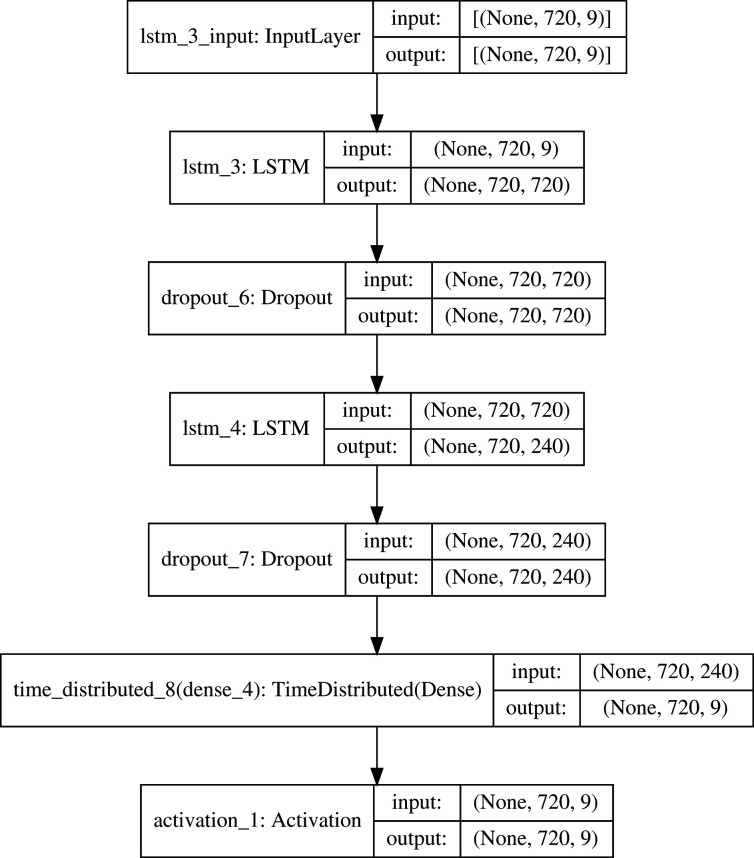
LSTM + encoder: an LSTM encoder layer attached to an LSTM decoder layer.

**Figure 14.**
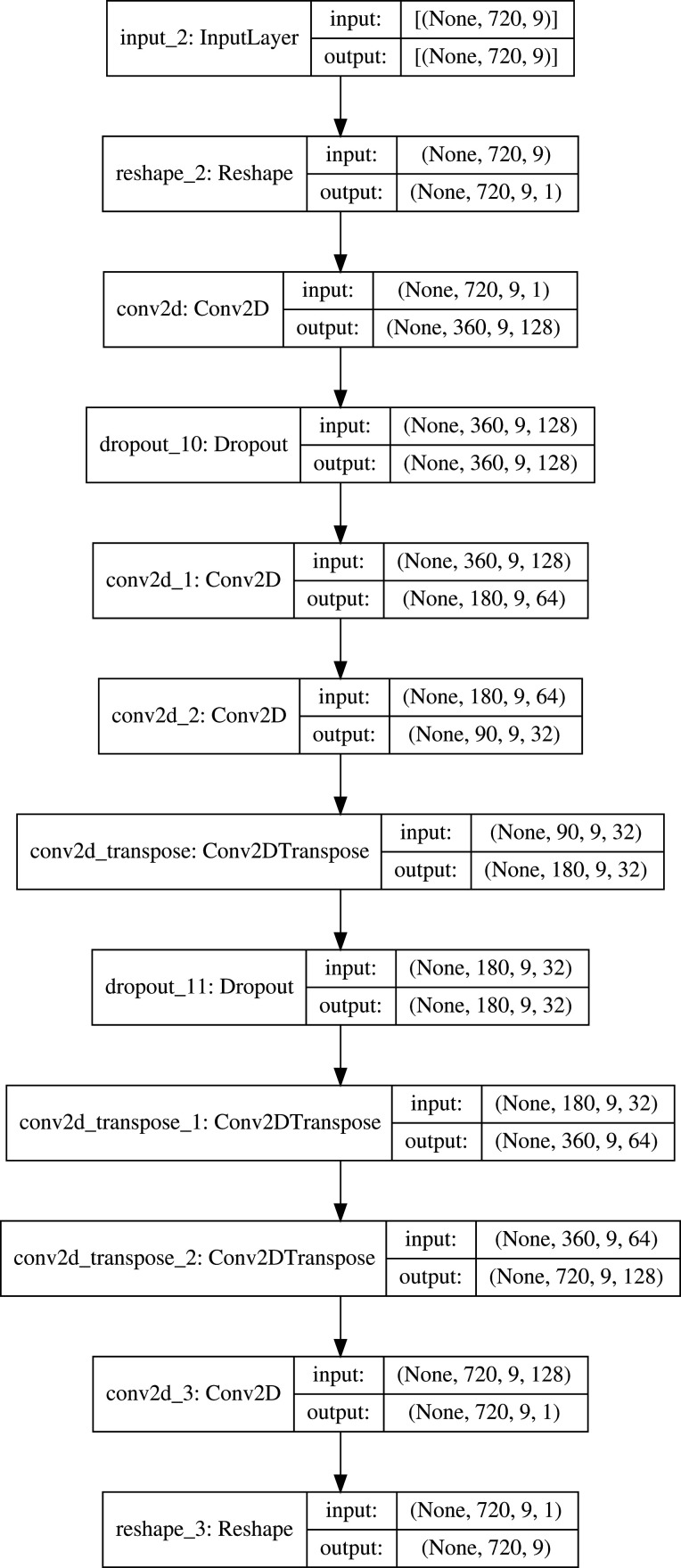
Autoencoder2D: an autoencoder with three two-dimensional convolution layers functioning as an encoder and three two-dimensional convolution layers functioning as the decoder.

**Figure 15.**
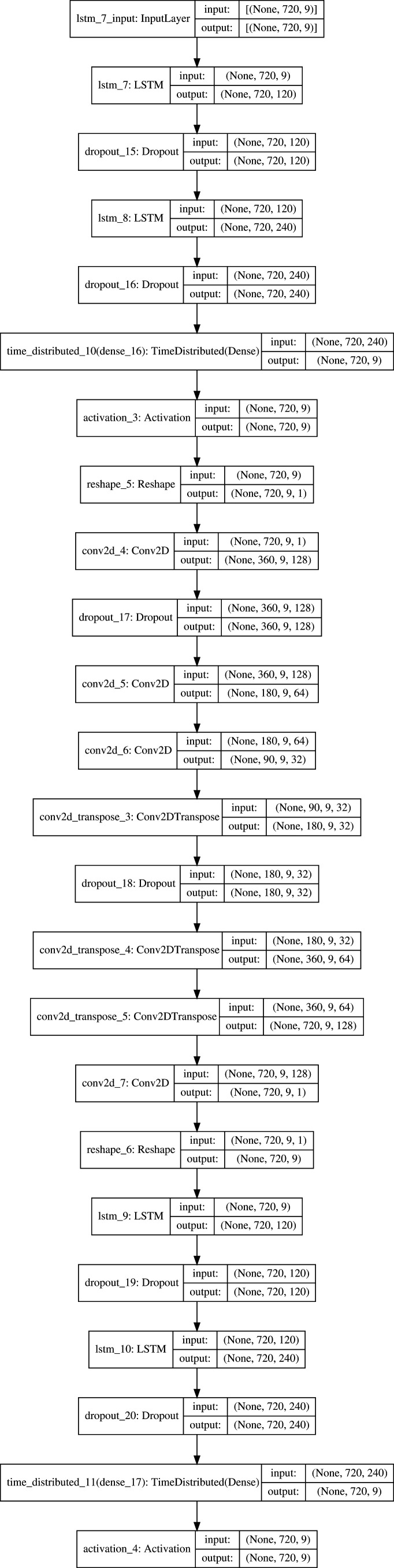
LSTM + autoencoder: this network is made of three blocks of layers in sequence. the first block is identical to the LSTM encoder–decoder described in figure 13, the second (middle) block is an autoencoder identical to the one in figure 14 and the third block is identical to the first one.

The classification results of both instances are presented as confusion matrices in figure 10. The average accuracies of the standard and the reconstructed model were calculated to be 48% and 52%, respectively. Therefore, on average the model trained with the reconstructed data performs slightly better than the model trained with the standard data. If we compare the two matrices, we can see that the gap-filling procedure, and therefore a presence additional data, does have an effect on the resulting accuracy of the ResNet model. Apart from the species *Abies alba* (label 3), all the other diagonal cells see an increase as a result of data imputation.

Nevertheless, the case of *Abies alba* can also be related to improvements in accuracy. From figure 10, we observe that in 15% and 45% of the cases, the standard model misidentifies it as *Fagus sylvatica* (label 1) and *Fraxinus excelsior* (label 6), respectively. However, the uncertainties are reduced correspondingly by 11% and 4% with the reconstructed model, while the misidentification of *Picea abies* as *Abies alba* increases. This change is what we would expect to see if we added more time-series samples to the dataset. *Picea abies* and *Abies alba* belong to the same species group while *Fagus sylvatica* does not. Therefore, we should expect the classifier to learn to distinguish first between gymnosperms and angiosperms and then among the species of the same group. Moreover, the increase of probabilities close to the diagonal at the cost of the off-diagonals can be seen as a general pattern, valid for all species. The result is less pronounced for the angiosperms because the sample size is much smaller in comparison with the gymnosperms.

Considering the relatively small amount of data available, which in addition is also imbalanced, we might expect that a poor performance of the imputation procedure would have a negative impact on the ResNet classifier. Our results show that this is not the case. On the contrary, we obtain a slight improvement in the model accuracy.

## 5. Conclusion and outlook

Complete and accurate data is a fundamental prerequisite for the analysis and exploitation of big data. However, more often than not, the data that we need is plagued with missing values, noise, drift and other unwanted artefacts, especially when it is in the form of a sequence that is continuously acquired by sensors in an unpredictable and noisy environment.

When dealing with data in the form of time series, it is always worthwhile to consider using an adequate deep neural network to replace any missing values with accurate estimates that go beyond simple interpolation. These machine-learning models also have the advantage that they can process multiple channels at the same time and that they can be implemented so that the entire process is automated and executed in real-time during data acquisition.

In the particular case of a multi-channelled time series consisting of measurements from different sensors, we have shown that a deep neural network composed of LSTM and one-dimensional CNN layers works best for imputation, with the most accurate prediction score. Although the gap-filling improved only slightly the accuracy of the classification model, the result is still remarkable. The original dataset was small to begin with, so that adding an extra 20% of reconstructed data had a substantial effect. Therefore, we expect that the accuracy of the classification algorithm would be negatively affected if the gap-filling algorithm did not perform well.

We are aware that we have not done an exhaustive research of test models, which is unrealistic and outside the scope of the study. Nevertheless, there are other machine-learning techniques that could prove to be viable candidates such as transformer architectures [26].

Considering the fact that it is relatively costly to install and operate the dendrometers on a large scale, to monitor the growth of more than just a few hundred trees, it would be very useful to determine how well the models represented in this paper would be able to reconstruct the entire SR signal, and not just a gap of missing data, solely from the environmental data channels. The implications of being able to reconstruct the tree stem growth signal from environmental data alone would be far-reaching. We would be able to monitor entire regions of forest in real time and perhaps study more closely how the trees as a large complex system react to climate change for example.

## Data Availability

Most of the data used in this work is available under the repository: https://doi.org/10.1111/nph.17552 [3].
